# Integrative miRNA-mRNA Profiling of Adipose Tissue Unravels Transcriptional Circuits Induced by Sleep Fragmentation

**DOI:** 10.1371/journal.pone.0037669

**Published:** 2012-05-21

**Authors:** Sina A. Gharib, Abdelnaby Khalyfa, Amal Abdelkarim, Bharat Bhushan, David Gozal

**Affiliations:** 1 Center for Lung Biology, Department of Medicine, University of Washington, Seattle, Washington, United States of America; 2 Division of Pulmonary and Critical Care Medicine, Department of Medicine, University of Washington, Seattle, Washington, United States of America; 3 Department of Pediatrics, Biological Sciences Division, The University of Chicago, Chicago, Illinois, United States of America; Nihon University School of Medicine, Japan

## Abstract

Obstructive sleep apnea (OSA) is a prevalent condition and strongly associated with metabolic disorders. Sleep fragmentation (SF) is a major consequence of OSA, but its contribution to OSA-related morbidities is not known. We hypothesized that SF causes specific perturbations in transcriptional networks of visceral fat cells, leading to systemic metabolic disturbances. We simultaneously profiled visceral adipose tissue mRNA and miRNA expression in mice exposed to 6 hours of SF during sleep, and developed a new computational framework based on gene set enrichment and network analyses to merge these data. This approach leverages known gene product interactions and biologic pathways to interrogate large-scale gene expression profiling data. We found that SF induced the activation of several distinct pathways, including those involved in insulin regulation and diabetes. Our integrative methodology identified putative controllers and regulators of the metabolic response during SF. We functionally validated our findings by demonstrating altered glucose and lipid homeostasis in sleep-fragmented mice. This is the first study to link sleep fragmentation with widespread disruptions in visceral adipose tissue transcriptome, and presents a generalizable approach to integrate mRNA-miRNA information for systematic mapping of regulatory networks.

## Introduction

Obstructive sleep apnea (OSA) is a highly prevalent disorder in adults and children [1]–[3] and associated with significant cognitive, metabolic, and cardiovascular morbidities [4]–[8]. OSA is characterized by recurrent collapse of the upper airway during sleep, leading to intermittent hypoxia and sleep fragmentation (SF). There is accumulating evidence that OSA is strongly linked to metabolic dysregulation, independent of obesity [9], [10]. Furthermore, fragmentation of sleep architecture is being recognized as an important contributor to OSA-related morbidities and can cause altered glucose homeostasis even in normal subjects [11]. SF may promote the adverse metabolic consequences of sleep apnea by perturbing normal visceral adipose tissue function and altering insulin sensitivity [12], [13], but the biological effects of SF on fat tissue are not known. Since visceral fat tissue depots are critical regulators of metabolism [14], [15], understanding the pathophysiologic consequences of SF on adipocyte biology is a crucial step in elucidating mechanisms linking OSA with metabolic dysregulation.

The advent of transcriptional profiling using mRNA microarrays has yielded important insights into the function of adipose tissue under various conditions [16]–[18]. More recently, microRNAs (miRNAs)—small, noncoding single strand RNAs abundant in eukaryotic cells—have emerged as central regulators of gene expression, exerting their influence through partial base-pairing with their target mRNAs that leads to their degradation or repression of translation [19], [20]. miRNAs are key effectors of many biological processes including adipocyte differentiation, metabolism, and insulin homeostasis [21]–[24]. A few studies have exploited miRNA profiling to define the transcriptional response of adipose tissue under normal and disease conditions [25]–[28], but no such efforts have been reported in OSA patients or in animal models of sleep apnea. The ability to simultaneously interrogate mRNA and miRNA gene expression from the same tissue is a promising venue for constructing gene regulatory networks in complex disorders, and a number of strategies for combining miRNA with mRNA have been proposed [29]–[32]. To our knowledge, this integrative approach has not been applied to investigate the effects of SF on the transcriptional circuitry of adipocytes. In this study, we induced SF in mice and developed a novel framework to mesh information from large-scale mRNA-miRNA profiling of visceral adipose tissue in order to systematically identify perturbed pathways within the context of their putative regulatory miRNAs. We validated our approach by demonstrating the functional relevance of a highly enriched module—“insulin regulation and diabetes”—by assessing glucose homeostasis and insulin resistance in sleep-fragmented mice.

## Materials and Methods

### Animal experiments

Male C57BJ/6L mice (Jackson Laboratory, Bar Harbor, Maine) of 10 weeks of age were used in this study. These mice were housed in 12-hour light/dark cycle (lights on at 7 a.m. to 7 p.m.) in constant temperature (26±1°C) with *ad libitum* access to food and water. At the end of the experimental period, the mice underwent brief anesthesia using carbon dioxide (1–2 min) and were euthanized immediately by cervical dislocation. Abdominal cavity was surgically opened and white (visceral) adipose tissue was dissected from the epididymis within <5 min, immediately frozen in liquid nitrogen, and stored at −80°C until RNA extraction. All protocols were approved by the Institutional Animal Care and Use Committee and are in close agreement with the National Institutes of Health *Guide in the Care and Use of Animals*. All efforts were made to minimize animal suffering and to reduce the number of animals used.

### Sleep fragmentation (SF)

Using a rodent model of sleep fragmentation recently developed and validated in our laboratory [33], [34], sleep was disrupted by the movement of an automated bar for the duration of exposure (6 h). This method prevents the need for human contact and intervention, introduction of foreign objects or touching of the animals during sleep, and is therefore superior to other existing methods of sleep disruption. To induce moderate to severe sleep fragmentation (mimicking patients with sleep apnea), we chose a 2-minute interval between each sweep, implemented during the light period (7 a.m. to 7 p.m.) with no activation of the sweeper during the night period (7 p.m. to 7 a.m.). Of note, two mice were housed in each sleep fragmenter cage to minimize isolation stress.

### RNA isolation

Total RNA and miRNA were isolated from the same visceral fat adipose tissues of each animal (SF: n = 8, control: n = 8). Total RNA was isolated using automated RNA extraction (Promega, Madison, WI) and DNase treated according to manufacturer's protocol. miRNA was isolated using miRNeasy Mini kit (Qiagen, Turnberry Lane, Valencia, CA.). The RNA quality and integrity were determined using the Eukaryote Total RNA Nano 6000 LabChip assay (Agilent Technologies) on the Agilent 2100 Bioanalyzer. The quality of miRNA was determined using Agilent Small RNA Kit according to the manufacture's protocol. Both total RNA and miRNA samples were quantified on a Nanodrop 2000 (Ambion, Austin, TX).

### Microarray experiments

#### mRNA microarrays

Whole-genome mouse Agilent microarrays were used to profile visceral fat gene expression during SF and control conditions. Purified total RNA (500 ng) was processed for labeling using the Low RNA Input Fluorescent Linear Amplification Kit (Agilent Technologies, Santa Clara, CA). Equal quantities of total RNA were labeled with each reaction containing 50 ng of total RNA and 2 µl (34 pg) of RNA spike-in control. Cyanine 3-labeled CTPs were obtained from PerkinElmer/NEN Life Sciences (Boston, MA). Agilent's Low Input Fluorescent Linear Amplification kit was used to generate cRNA for one color as we have previously described [35]. The quality of each cRNA sample was evaluated using 2100 Bioanalyzer (Agilent Technologies, Santa Clara, CA). Each sample was hybridized to an Agilent oligonucleotide microarray for a total of 16 independent experiments. The microarray slides were scanned using Agilent dual-laser Microarray Scanner and the digitized images were acquired and processed using Agilent Feature Extraction (FE) software v.9.5. One of the mRNA microarray experiments (in the control group) failed our hybridization quality assessment and was excluded from further analysis. Background-subtracted intensities were normalized using the quantile method across all remaining 15 microarray experiments.

#### miRNA microarrays

Each sample was prepared according to the Agilent's miRNA (627 mouse miRNAs and 39 mouse viral miRNA) using the one-color technique in accordance with the manufacturer's instructions. The samples were profiled on the Agilent Mouse miRNA Microarray (Agilent Technologies), consisting of 60-mer DNA probes synthesized in situ that represent 620 mouse miRNA and 39 viral miRNAs from the Sanger database (Version 10.1), using the one-color technique in accordance with the manufacturer's Instructions. Total RNA including enriched miRNA (100 ng) was dephosphorylated with calf intestine alkaline phosphatase (GE Healthcare Europe GmbH), denatured with dimethyl sulfoxide, and labeled with pCp-Cy3 using T4 RNA ligase (GE Healthcare Europe GmbH). The labeled RNAs were hybridized to Mouse miRNA Microarrays Agilent Technologies (Santa Clara, CA). After hybridization and washing, the arrays were scanned with an Agilent microarray scanner using high dynamic range settings as specified by the manufacturer. Microarray results were extracted using Agilent Feature Extraction software (v9.5.3.1) [36]. Undetected probes were excluded from further analysis. Background-subtracted intensities were normalized for detected miRNA probes using the quantile method across all 16 microarray experiments.

### Microarray data analysis

#### mRNA microarrays

To determine enriched biologic processes, we applied gene set enrichment analysis (GSEA) to the visceral fat mRNA microarray dataset of mice exposed to SF (n = 8) and controls (n = 8) [37]. We performed random gene set permutation (n = 2000) using approximately 3200 curated gene sets and 1400 Gene Ontology annotations. We chose a false discovery rate (FDR, *Q*-value) threshold of <5% for significance. Since even using a strict FDR cutoff, many pathways and genes were significantly enriched, we limited our analysis to subsets of differentially expressed genes within each gene set that were the primary drivers of the enrichment score, known as the leading edge [38]. Furthermore, gene sets involved in similar functions such as “insulin regulation” and “diabetes pathways” or “mitochondrion” and “oxidative phosphorylation” were combined.

#### miRNA microarrays

We identified differentially expressed miRNAs in visceral fat tissue of SF and control mice using a Bayesian implementation of the parametric *t*-test combined with FDR correction using *Q*-value (cutoff<5%). Gene targets for differentially expressed miRNAs were computationally predicted using the miRanda algorithm [39], [40] as implemented in the MicroCosm Target web resource (http://www.ebi.ac.uk/enright-srv/microcosm/htdocs/targets/v5/) [41].

#### Schema for integrating mRNA-miRNA data

**Figure 1:**
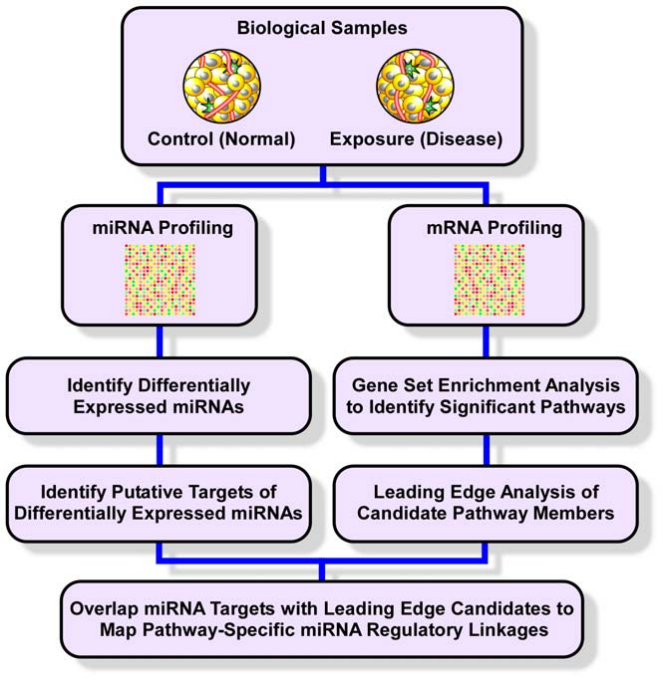
Outline of a general approach for integrating mRNA-miRNA data. Concurrent miRNA and mRNA profiling of the same tissue under control and exposure conditions is performed. The mRNA information is processed using gene set enrichment analysis with further refinements based on leading edges of enriched biologic modules. Meanwhile, differentially expressed miRNAs as well as their computationally-derived targets are also identified. These data are then merged based on anti-correlated expression of leading edge genes and their corresponding putative miRNAs.

Figure 1 outlines our general model for mRNA-miRNA data integration. This approach is based on two principles. First, we assume that the expression of a given miRNA is anti-correlated with the mRNA expression of its targets. This is a widely accepted and experimentally verified supposition [42]. Second, unlike other published methods based on tests identifying differential expression of individual genes, we select enriched gene sets from the mRNA microarray experiment using GSEA. Note that other proposed pathway-centric methodologies can be substituted for GSEA in this step [43], [44]. The rationale for this approach is the recognition that biologic processes result from coordinated activation of coherent sets of genes known as modules [45], [46]—a paradigm ideally captured thorough pathway enrichment analysis. The final step of this procedure involves systematic linkage of differentially expressed miRNAs to their corresponding pathways based on the presence of predicted target genes within enriched modules.

#### Network analysis

A genetic network was constructed for the enriched “insulin regulation and diabetes pathways” module based on experimentally verified gene product interactions derived from Ingenuity [47] and STRING [48] knowledgebases. Insulin was incorporated within the network to highlight its central relationship with the other nodes.

### Quantitative RT-PCR (qPCR)

qPCR analysis was performed for selected mRNAs and miRNAs using ABI PRISM 7500 System (Applied Biosystems, Foster City, CA). The same total RNA was used for both microarray and qPCR experiments. To confirm candidate mRNAs, cDNA was synthesized using one microgram of total RNA using a High-Capacity cDNA Archive Kit (Applied Biosystems, Foster City, CA). Ribosomal 18S rRNA, was used as a reference gene to normalize the expression ratios. All experiments were performed in triplicates. The cycle number (C_t_) values were averaged and the difference between the 18S C_t_ and the gene of interest C_t_ were calculated to calculate the relative expression of the gene of interest using the 2^−ΔΔCt^ method [49]. The results are presented as fold change (SF relative to control conditions).

miRNAs selected for confirmation were reverse transcribed with looped microRNA-specific reverse transcriptase (RT) primers (Applied Biosystems, Foster City, CA) using the TaqMan microRNA mouse assay according to the manufacturer's protocol. Briefly, RT reactions were performed in a volume of 15 µl, and each reaction contained 10 ng of enrich miRNA. RT reactions were performed on a GeneAmpPCR System 9600 (Applied Biosystems, Foster City, CA). Reactions without addition of reverse transcriptase were performed alongside cDNA synthesis of each sample and used in subsequent procedures to control for potential genomic DNA contamination. All TaqMan assays were run in triplicate on an ABI Prism 7500 using TaqMan Universal PCR Master Mix II without UNG (Applied Biosystems, Foster City, CA). The qPCR results were normalized against an internal control (RNU6), and then expressed as fold changes (SF relative to control conditions).

### Metabolic assessments

Metabolic parameters were measured in a different group of control (n = 8) and SF (n = 7) mice following 6 hours of fasting (with *ad libitum* access to water). Blood glucose and insulin levels were measured immediately before glucose injection and the homeostasis model assessment (HOMA) insulin resistance (IR) equation (HOMA-IR index) was calculated [50]. For glucose tolerance test (GTT), mice were injected with (2 g/kg) of D-glucose (Sigma Chemical Co., St. Louis, MO) intra-peritoneally. Blood samples were collected from lateral tail vein by puncture at 0, 15, 30, 60 and 120 min following the glucose injection. Glucose levels were measured using a glucometer (One Touch Ultra, Lifescan). Triglyceride levels were measured in an independent group of control mice (n = 5) and animals exposed to 3 weeks of SF (n = 5). Mice were fasted for 3 hours prior to blood collection. Plasma triglyceride levels (mg/dl) were measured using Infinity kits (Thermo Fisher Scientific, Waltham, MA) according to the manufacturer's protocol.

### Statistical analysis

Two-tailed Mann-Whitney test with unequal variance was used to compare HOMA-IR and plasma triglyceride levels between SF and control mice (GraphPad Prism version 5, San Diego, CA). The GTT time course experiment was analyzed using two-way ANOVA with time and exposure (SF, control) as variables. The ANOVA *P*-value reported corresponds to the significance of exposure effect. All data are shown as mean ± SEM.

## Results

### SF elicits differential expression of miRNAs in visceral adipose tissue

**Figure 2:**
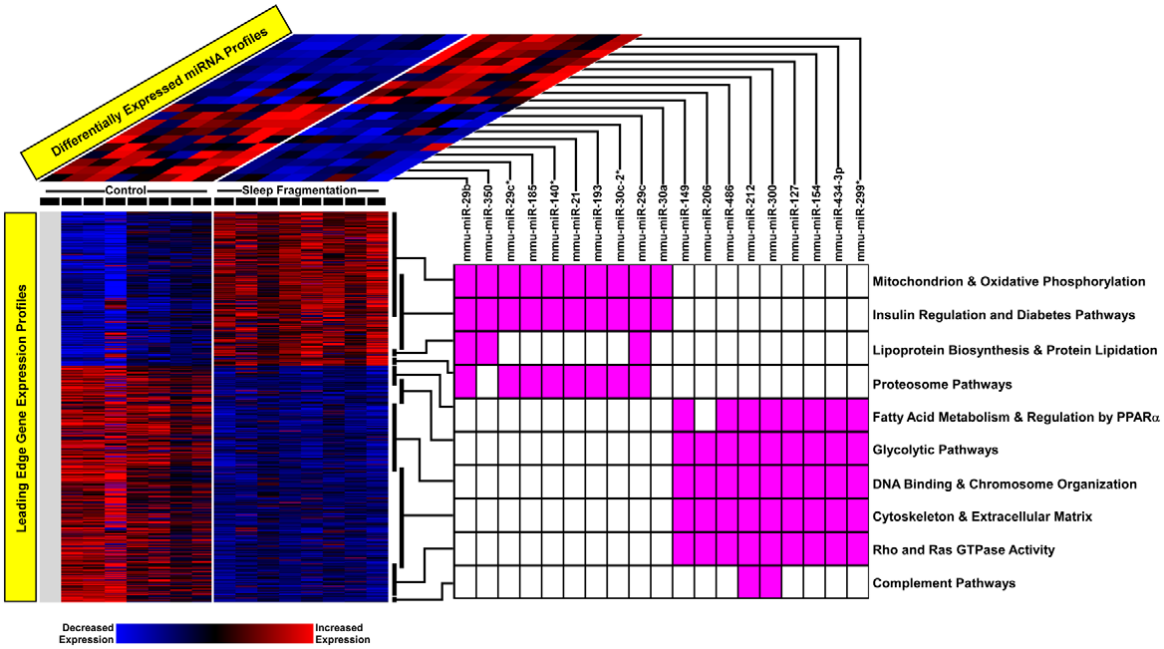
Integrated mRNA-miRNA analysis of visceral fat tissue transcriptome during SF. Heatmaps depict gene expression profiles of enriched pathways and differentially expressed miRNAs in response to SF. The miRNAs are linked to their respective modules based on whether these pathways harbor target genes. Note that one mRNA microarray experiment was excluded due to poor hybridization (gray column).

We identified 19 differentially expressed miRNAs (9 up-regulated, 10 down-regulated) in response to SF in visceral fat cells using a *Q*-value cutoff <5% (Figure 2). We confirmed the differential expression of several candidate miRNAs using qPCR as presented in Table 1.

**Table 1:** qPCR confirmation of selected differentially expressed miRNAs.

	Microarray		qPCR	
	*P*-value	Fold	*P*-value	Fold
mmu-miR-29b	5.4×10^−4^	−1.68	1.0×10^−2^	−1.59
mmu-miR-350	2.4×10^−4^	−1.54	4.0×10^−3^	−0.46
mmu-miR-185	3.9×10^−3^	−1.46	2.0×10^−3^	−1.47
mmu-miR-154	2.5×10^−7^	2.39	2.0×10^−3^	2.79
mmu-miR-300	1.4×10^−6^	1.95	3.0×10^−3^	2.16
mmu-miR-434-3p	2.8×10^−7^	2.42	2.0×10^−3^	3.79

### Exposure to SF alters the transcriptional profile of visceral adipocytes and is associated with enrichment of specific pathways

We applied GSEA to mRNA probesets matching to unique genes and demonstrated that SF induced profound transcriptional perturbations in fat tissue (Figure 2). We confirmed our findings for select candidate genes using qPCR (Table 2). Subsequent leading edge analysis identified two distinct patterns of differential gene expression, each associated with enrichment of select gene sets in mice exposed to SF or controls (enrichment *Q*-value<5%). Biologic modules comprised of up-regulated genes in response to SF included “mitochondrion and oxidative phosphorylation”, “insulin regulation and diabetes pathways”, “lipoprotein biosynthesis and protein lipidation” and “proteasome pathways”. Modules enriched in genes down-regulated during exposure to SF included “fatty acid metabolism and regulation by PPARα”, “glycolytic pathways”, “DNA binding and chromosomal organization”, “cytoskeleton and extracellular matrix”, “Rho and Ras GTPase activity” and “complement pathways”.

**Table 2:** qPCR confirmation of selected differentially expressed genes.

	Microarray		qPCR	
	*P*-value	Fold	*P*-value	Fold
Mlx	6.6×10^−3^	1.46	5.4×10^−3^	1.84
Gja4	2.2×10^−6^	2.11	1.0×10^−3^	2.79
Mt1	5.6×10^−6^	2.69	3.0×10^−3^	2.16
Srpk2	4.1×10^−6^	2.40	2.0×10^−3^	3.79
Cxcl13	1.7×10^−6^	−8.58	5.0×10^−3^	−3.67
Aldh2	1.3×10^−8^	−5.79	3.0×10^−3^	−4.22
Acaca	5.6×10^−6^	−3.99	2.0×10^−3^	−3.78

### miRNA-mRNA data integration identifies putative regulators of SF-induced pathways

As outlined in Figure 1 and discussed in the Methods section, we systematically linked differentially expressed miRNAs with gene members of enriched pathways based on anti-correlated expression levels. Figure 2 summarizes our results and highlights putative regulatory relationships between miRNAs and their corresponding biologic pathways in visceral adipose tissue of sleep-fragmented mice. All differentially expressed miRNAs were associated with at least one enriched pathway and in many instances interacted with multiple modules.

### Network analysis reveals control sites of a representative enriched pathway

**Figure 3:**
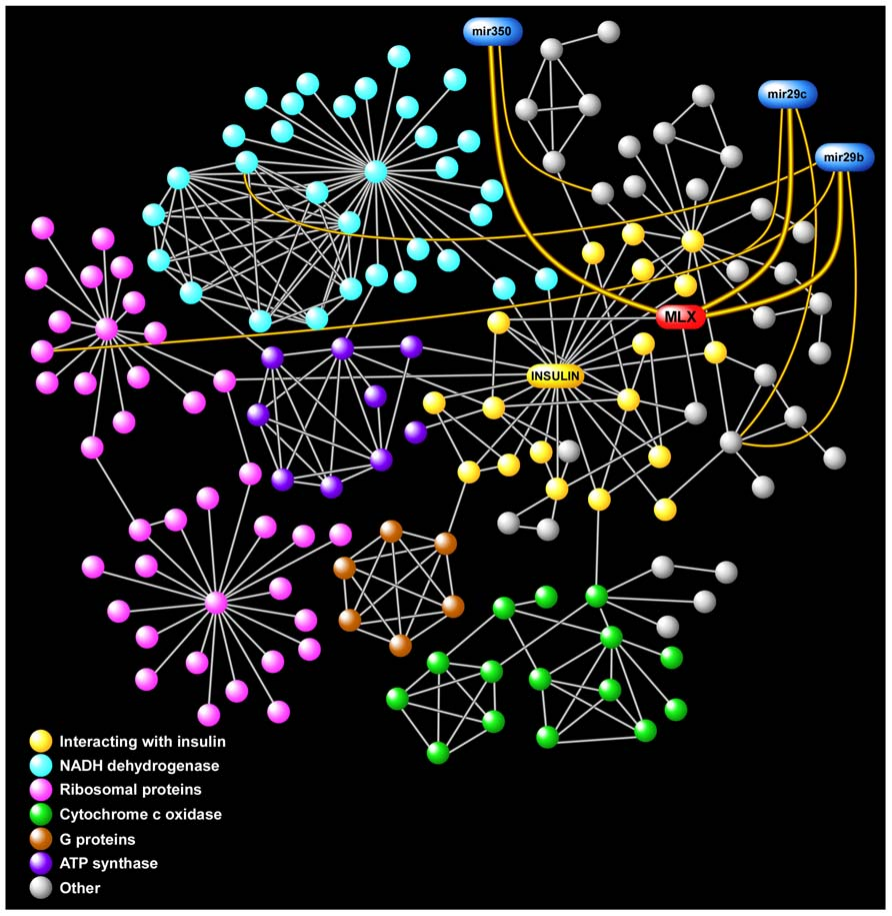
Network analysis of the “insulin regulation and diabetes” pathway. Members of this enriched module were linked together based on verified gene product interactions. Several distinct motifs were identified (shown in different colors). As expected, insulin assumed a central position in this network. One upregulated candidate, MLX (red), was the putative target of multiple downregulated miRNAs (blue) and is postulated to be a critical controller of visceral adipocytes' metabolic response to SF. Note that several other downregulated miRNAs interacted with members of this network, but are not shown in order to improve clarity.

To further elucidate relationships among members of gene sets, we constructed a gene product interaction network, or interactome, for the “insulin regulation and diabetes” module. As depicted in Figure 3, this interactome—like many biologic networks [51]—was characterized by a few densely connected hubs and several distinct motifs representing key functional units including NADH dehydrogenase, ribosomal activity, ATP synthase, and cytochrome c oxidase. Importantly, many members of this module interacted with insulin, highlighting the expected central role played by this molecule. Superimposed on the “insulin regulation and diabetes” interactome were differentially expressed miRNAs postulated to regulate its biologic function. We observed that a member of this module, MAX-like protein X (MLX), interacted with glucagon (GCG)—a central regulator of glucose homeostasis—and was the most highly connected putative target of miRNAs in the network, being linked to mmu-miR-29b, mmu-miR-29c, and mmu-miR-350 (Figure 3). We confirmed the anti-correlated expression pattern of two of these miRNAs and *Mlx* using qPCR (Tables 1 and 2). Our findings implied that since *Mlx* was a differentially upregulated gene and the putative target of multiple downregulated miRNAs, it may play an important regulatory role in the “insulin regulation and diabetes” module—a prediction that is supported by recent reports [52], [53].

### Functional validation of dysregulated glucose and lipid homeostasis following SF

**Figure 4:**
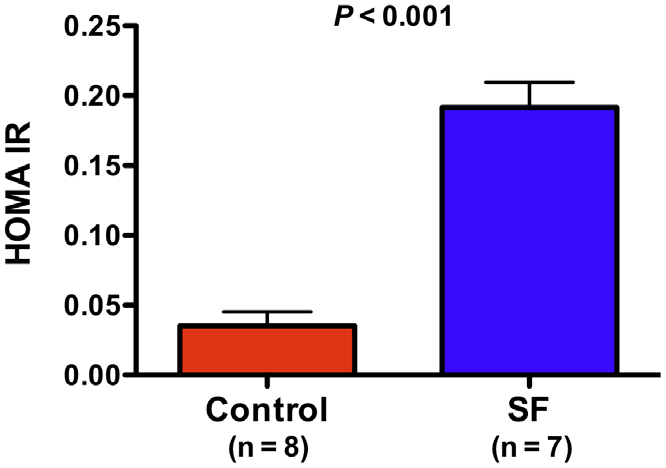
Acute sleep fragmentation causes dysregulation in glucose homeostasis as demonstrated by the development of insulin resistance in SF mice using the homeostatic model assessment (HOMA-IR). Data are presented as mean ± SEM.

**Figure 5:**
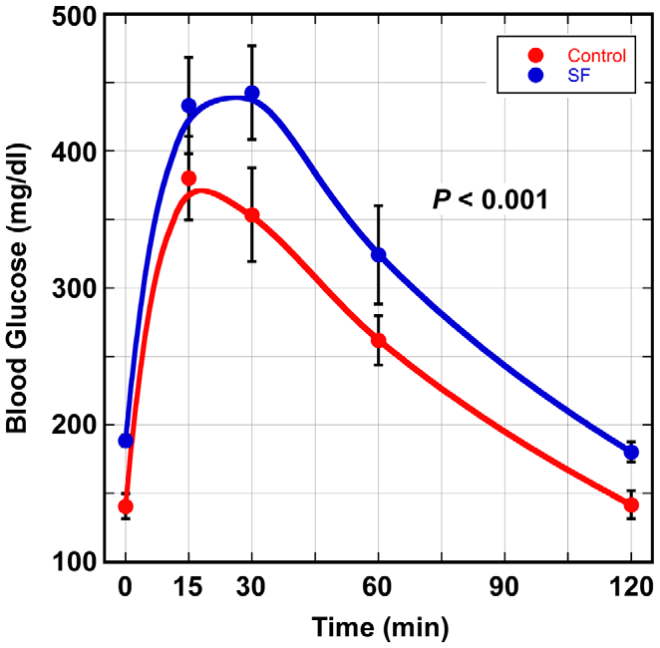
Reduced glucose tolerance in mice exposed to SF compared to control animals. Mice (n = 8 control, n = 7 SF) were injected with of D-glucose (2 g/kg) intra-peritoneally and glucose levels measured at 0, 15, 30, 60 and 120 min following injection. *P*-value was based on two-way ANOVA and corresponds to the significance of exposure (glucose) effect. All data are shown as mean ± SEM.

**Figure 6:**
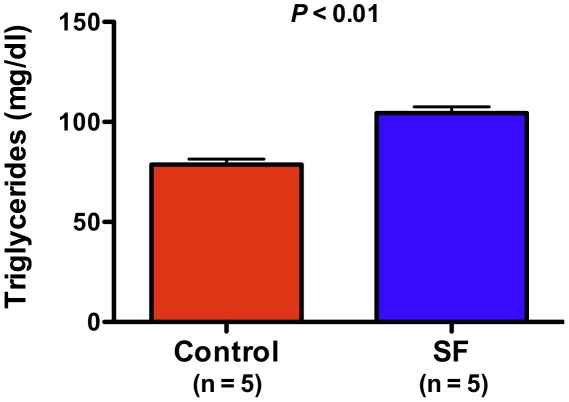
Chronic sleep fragmentation is associated with elevated circulating triglycerides. Mice were subjected to 3 weeks of SF (n = 5) or used as controls (n = 5). Fasting plasma triglyceride levels were measured and are presented as mean ± SEM.

Since our computational analyses highlighted perturbations in “insulin regulation and diabetes” gene set during exposure to SF, we proceeded to compare metabolic measures of glucose homeostasis in SF and control mice. As shown in Figures 4 and 5, animals exposed to 6 hours of SF developed significant insulin resistance and impairment of glucose tolerance. Another enriched module, comprised of downregulated genes after SF, was “fatty acid metabolism and regulation by PPARα” (Figure 2). We have observed systemic disturbances in lipid profiles of mice after chronic exposure to SF (unpublished data) and therefore measured fasting plasma triglyceride levels in mice following 3 weeks of SF. As depicted in Figure 6, mice subjected to chronic SF had significant elevation of their triglyceride levels, implying dysregulated lipid metabolism.

## Discussion

In this study, we report on the first systematic effort to map the regulatory transcriptional landscape of visceral adipocytes in response to sleep fragmentation—a key pathophysiologic event in sleep apnea. To achieve this aim, we developed a framework for integrating mRNA-miRNA expression profiles based on gene set enrichment and network analyses. Our results demonstrated that exposing mice to SF induced widespread alterations in their fat cell transcriptome and was associated with enrichment of distinct pathways.

Although obstructive sleep apnea is associated with insulin resistance, impaired glucose tolerance, and type 2 diabetes [54]–[56], the role of SF in contributing to these metabolically dysfunctional states is not known. Our findings suggest that SF promotes metabolic disturbances by perturbing regulatory networks in visceral adipose tissue—including those involved in glucose homeostasis. Leveraging information based on known gene product relationships allowed us to fine-map putative interactions in the enriched “insulin regulation and diabetes” module, and identify potentially important metabolic effectors induced by SF. One such candidate, MLX, is a component of a heterodimeric transcription factor that is a master sensor of cellular energy status and regulator of glucose metabolism [52], [53], [57]. In our animal model of SF, MLX was upregulated in visceral adipocytes and was a putative target of several differentially expressed miRNAs. While the biological roles of miRNAs in adipose tissue remain mostly unknown, reduced circulating levels of one of the MLX-regulating miRNAs—mmu-mir-29b—has been associated with the development of type 2 diabetes [58], and the mmu-mir-29 family may be involved in insulin signaling [59]. We confirmed that following acute exposures to SF, mice develop insulin resistance and glucose intolerance. Future work will be required to determine the molecular mechanisms mediated by candidate genes and miRNAs highlighted from our analysis that lead to the observed metabolic dysregulation. There is increasing evidence that circulating levels of miRNAs may be useful diagnostic and prognostic biomarkers for common human disorders including obesity [60], diabetes [58] and cardiovascular diseases [61], although no study to date has investigated a role for miRNAs in sleep apnea.

In addition to the “insulin regulation and diabetes” gene set, we identified several other enriched modules comprised of differentially upregulated genes in response to SF, including “mitochondrion and oxidative phosphorylation” and “proteasome” gene sets. These finding are consistent with our recent report identifying these pathways as highly activated in visceral adipose tissue of mice exposed to intermittent hypoxia [62]. Since intermittent hypoxia and SF represent two critical perturbations in sleep apnea, the overlapping of enriched gene sets implies that at the functional pathway level, these insults promote similar alterations in adipocyte biology.

It is important to note that while adipose tissue is a critical regulator of glucose metabolism, many other organs such as the liver, pancreas, hypothalamus, and skeletal muscle are also important contributors. It is therefore highly likely that SF also alters the transcriptional response within these tissues. Comprehensive profiling of other metabolic regulators was not within the scope of this project and represents a future direction. Nevertheless, adipose tissues in general, and visceral fat in particular, are widely regarded as the orchestrator of lipid storage and metabolism—primarily in the form of triacylglycerol (triglyceride). To assess whether SF is associated with adipose tissue dysfunction, we measured plasma triglyceride levels in mice subjected to 3 weeks of SF and found that chronic sleep disruption resulted in significant elevation of circulating triglycerides (Figure 6). Consistent with this observation, our computational analyses had identified “fatty acid metabolism and regulation by PPARα” as an enriched gene set with most of its members down-regulated in response to SF (Figure 2). Peroxisome proliferator-activated receptor-alpha (PPARα) is a key controller of lipid metabolism [63] and can be pharmacologically activated by fibrates—a class of drugs used clinically to lower triglyceride level. Our findings suggest that this pathway may also represent a potential therapeutic target for sleep apnea-associated dyslipidemia.

In this work, we outlined a simple strategy to integrate data from concurrent mRNA and miRNA profiling of the same tissue. Unlike previous methods based on gene-level statistics, we implemented a gene set enrichment approach because most biologic processes result from coordinated activation of many genes [45]. Importantly, since a given miRNA has multiple targets, our methodology is ideally suited to capture the role of these regulatory molecules in the context of functionally relevant pathways. Furthermore, this approach is quite flexible (e.g., other enrichment algorithms or metrics for differential gene expression can be substituted) and is generalizable to any *in vivo* or *in vitro* experimental system.

This study has several limitations. Exposure to SF was short (6 hours); future work is required to investigate the chronic effects of SF on adipose tissue and metabolism. Although we confirmed differential expression of several candidates using qPCR, we did not measure differences at the protein level. Furthermore, miRNA targets were identified based on computational predictions and not functionally validated. Finally, our enrichment and network analyses represented the current state of knowledge. Since many new relationships between gene products and pathways will be discovered in the future, the presented network analyses capture a subset of the biologic processes activated in this model.

In conclusion, we demonstrate that a short exposure to sleep fragmentation perturbs the transcriptional circuitry of visceral adipocytes and induces metabolic dysregulation in mice. We propose a general strategy to integrate whole-genome mRNA and miRNA profiling, and exploit this method to systematically map genetic networks in fat tissue and identify pathways induced by SF in the context of regulatory miRNAs.
